# Epistatic effects of *IGHG* and *FCGRIIB* genes on the development of Alzheimer’s disease in African Americans

**DOI:** 10.1007/s00251-024-01358-4

**Published:** 2024-11-04

**Authors:** Janardan P. Pandey, Paul J. Nietert, Aryan M. Namboodiri, David A. Bennett, Lisa L. Barnes

**Affiliations:** 1 Department of Pharmacology & Immunology, Medical University of South Carolina, Charleston, SC 29425-2230 USA; 2 Department of Public Health Sciences, Medical University of South Carolina, Charleston, SC USA; 3 Rush Alzheimer’s Disease Center, Rush University Medical Center, Chicago, IL USA

**Keywords:** Alzheimer’s disease, Heritability, GM (γ marker), *FCGRIIB*, *HLA-DRB1*, *PILRA*

## Abstract

Genome-wide association studies (GWAS) of Alzheimer’s disease (AD) have identified a large number of susceptibility genes, but most of AD heritability remains unexplained, implying the existence of additional genes. Furthermore, the majority of the GWAS have been conducted in people of European descent, and the genes important for AD susceptibility in people of African descent have been underexplored. In this hypothesis-generating prospective cohort study, we genotyped 191 African Americans (AAs) from three longitudinal cohorts on aging for the IgG3 allotype GM6, which is expressed exclusively in people of African descent, and assessed its interaction with *IGHG*, *FCGRIIB*, and *HLA-DRB1* genes. Cox proportional hazards modeling showed that GM6 by itself was not significantly associated with AD development. However, there was evidence of epistatic interaction: The risk of developing AD associated with GM6 positivity was significantly different (*p* = 0.0098) in non-GM17/GM17 participants compared with GM 17/GM17 participants. Specifically, in non-GM17/GM17 participants, the risk of AD was over fourfold higher in GM6-positive participants compared with GM 6-negative participants (HR = 4.63). Similarly, risk of developing AD associated with GM6 positivity was marginally different in non-FCGRIIB TT participants compared with FCGRIIB TT participants. In non-FCGRIIB TT participants, the risk of developing AD was over twofold higher in GM6-positive participants compared with GM6-negative participants (HR = 2.44). This is the first report suggesting that immunoglobulin GM allotypes might play a role in AD etiology among AAs; however, since this was largely a hypothesis-generating study, replication in larger cohorts would be required to confirm this finding.

## Introduction

Late-onset AD is the leading cause of dementia in adults. Although the etiology of the disease remains unclear, both genetic and environmental factors have been implicated in AD pathogenesis. Genome-wide association studies (GWAS) of AD have identified numerous susceptibility genes, but only a fraction of AD heritability can be attributed to these genes, implying the existence of additional risk-conferring genes. Furthermore, the majority of the GWAS of AD have been conducted in people of European descent, and the genes important for AD susceptibility in people of African descent—who have a higher risk of developing the disease—have been underexplored.

Despite their name, the GWAS do not evaluate a major gene complex of the immune system—GM (γ marker) allotypes encoded by three highly homologous immunoglobulin heavy chain G (*IGHG1*, *IGHG2*, *IGHG3*) genes on chromosome 14 (Oxelius and Pandey 2013). GM genes are not included in the current genotyping platforms (Warrender and Kelton 2020; Pandey 2010a, b). The extensive homology of the GM allotype-encoding chromosomal region makes it unamenable to high-throughput genotyping technology, which could have contributed to their exclusion from major genotyping arrays. Using candidate gene approaches, we, and others, have shown GM gene involvement in the immunobiology of many human diseases, including neurological diseases, such as multiple sclerosis, neuroblastoma, glioblastoma, schizophrenia, AD, and Parkinson’s disease (Buck et al. 2013; Pandey et al. 1981, 2014a, 2016, 2020, 2022; Salier et al. 1981; Morell et al. 1977).

In AD, using a nested case–control design, particular GM allotypes have been shown to be risk factors for the development of the disease in a Swedish cohort (Pandey et al. 2020) but their possible role in AD susceptibility in people of African descent is not known. Expression of GM allotypes has been shown to vary significantly across population groups. The GM 6 allotype—characterized by the simultaneous presence of serine and glutamic acid at positions 44 and 98 of IgG3 antibody, respectively—is expressed almost exclusively in people of African descent (Lefranc and Lefranc 2023). The aim of the present investigation was to determine whether GM 6 genotypes—individually and/or epistatically with other GM genotypes—were associated with AD development in an AA cohort.

Additionally, we aimed to determine whether GM 6 genotypes interacted with three other candidate genes—*HLA-DRB1* rs9271192, paired immunoglobulin type 2 receptor alpha-*PILRA* rs1859788, and *FCGRIIB* rs1050501—and epistatically contributed to AD development. *HLA-DRB1* has been shown to be associated with AD in many studies (Lu et al. 2017). *FCGRIIB* contributes to the inter-neuronal accumulation of amyloid-β (Aβ), a hallmark of AD (Kam et al. 2013). *FCGRIIB* rs1050501 is associated with AD in AA participants (Pandey et al. 2021). *PILRA* is a co-receptor for the cell entry of herpes simplex virus type 1 (HSV1), a risk factor for AD development (Itzhaki 2018).

## Materials and methods

### Participants and procedures

This investigation used a prospective cohort design and archived DNA samples and data from three longitudinal cohorts on aging in the USA: The Minority Aging Research Study (MARS), The Rush Memory and Aging Project (MAP), and The Religious Orders Study (ROS), which have been described in detail elsewhere (Barnes et al. 2012; Bennett et al. 2018). DNA from 191 self-identified AA participants was available for this investigation (MARS, *n* = 32; MAP, *n* = 157; ROS, *n* = 2). The participants from these cohorts were generally similar with respect to baseline age (MARS mean ± SD 72.8 ± 5.9; MAP 70.8 ± 6.2; ROS 73.6 ± 3.0) and years of follow-up (MARS 11.5 ± 5.0, MAP 11.5 ± 6.3, ROS 10.0 ± 2.8), with modest differences in sex (MARS 27% male, MAP 19% male, ROS 50% male), proportion developing AD (MARS 26%, MAP 41%, ROS 50%), and proportion dying during follow-up (MARS 30%, MAP 22%, ROS 100%). Three of the participants were believed to have AD at their baseline assessment, but none of these was included in the analyses of time to development of AD. The study was approved by the Institutional Review Boards of Rush University Medical Center and the Medical University of South Carolina. All participants signed an informed consent and a repository consent to allow their data to be shared. Cognitive impairment and Alzheimer’s dementia were diagnosed as reported (Bennett et al. 2002, 2006a, b).

### Genotyping

#### GM 6

An 895-base pair fragment of *IGHG3* gene that includes the location of GM 6 was amplified by PCR, using the following primer pair combinations (Calonga-Solís et al. 2019).

- Forward: 5′ AGG ACA GGT GCC CTA GAG TG 3′
- Reverse: 5′ CGT GCC AAG CAT CCT CG 3′

The PCR-amplified product was sequenced by Sanger sequencing, using the following primers:

- Forward: 5′ CTG CAT CCA GGG ACA GGT3′
- Reverse: 5′ ATC CTC GCG CGA CCC 3′

#### GM 3, 17, GM 23

IgG1 markers GM 3 and 17 (arginine to lysine) and IgG2 markers GM 23 − and 23 + (valine to methionine) were previously genotyped by a TaqMan® genotyping assay from Applied Biosystems Inc.

#### HLA-DRB1 rs9271192

*HLA-DRB1* SNP rs9271192 (A > C) was previously determined by a custom-designed rhAMP® SNP genotyping assay from Integrated DNA Technologies Inc.

#### *FCGRIIB* and *PILRA*

*FCGRIIB* (rs1050501 C/T) and *PILRA* (rs1859788 A/G) genotyping was done previously by real-time PCR detection, using custom-designed TaqMan® genotyping assays from Applied Biosystems Inc. (Foster City, CA).

### Statistical analyses

Since the GM 6 + / + genotype was relatively uncommon in this cohort, those with GM 6 + / + (*n* = 10) were combined with those with GM 6 + / − (*n* = 54) and classified as “GM 6 positive” participants; participants with the GM 6 − / − genotype were considered “GM 6 negative.” Associations between GM 6 and participant characteristics were made using the Wilcoxon rank sum tests (for continuous variables) and Fisher’s exact tests (for categorical variables). Association between GM 6 and time to development of AD was assessed using Cox proportional hazards models, which accounted for mortality and loss to follow-up. First, time to development of AD was modeled as a function of GM 6 status, while controlling for key covariates (baseline age, sex, years of education, *APOE*-ε4 carrier status). Next, for each of the other candidate genes, we examined (in separate Cox models) whether there was a significant main effect of the other candidate gene or a significant interaction between GM 6 and the candidate gene, after adjusting for covariates. In each of these models, participants with two copies of the candidate gene’s minor allele were combined with the heterozygous group to ensure that subgroup sample sizes were sufficient. For all models, the proportionality assumption was verified. As this was largely a hypothesis-generating study, initially no adjustment was made for multiple comparisons (Bender and Lange 2001); however, we also note which findings remain statistically significant after correcting for multiple comparisons using a false discovery rate (FDR) approach (Benjamini and Hochberg 1995). Analyses were conducted using SAS v9.4 (SAS Institute, Cary, NC).

## Results

Table 1 presents the baseline demographic characteristics for the study population and follow-up data regarding death and development of AD, stratified by the GM 6 genotype. No significant differences in these variables were noted between the GM 6-positive participants (GM6 + / − or GM6 + / +) and those who were negative for this determinant (GM6 − / −). Table 2 presents the distribution of GM 3/17, GM 23, *HLA DRB1*, *FCGRIIB*, and *PILRA* genotypes in AA participants, stratified by the GM 6 genotype. Significant (*p* < 0.05) differences between GM 6-carrying and non-GM 6-carrying participants were noted for the GM 3/17 and GM 23 loci; neither of these *p*-values, however, was significant after the FDR correction for multiple comparisons.

**Table 1:** Descriptive statistics for the study population (*n* = 191 African American participants), stratified by GM 6 genotype

Variable	Statistic	GM 6 − / − ( n = 127)	GM 6 + / − or GM 6 + / + ( n = 64)
Age at baseline (y)	Mean (SD)	72.3 (5.8)	72.6 (6.1)
Sex	*n*, % male	35 (27.6)	15 (23.4)
Education (y)	Mean (SD)	14.8 (3.6)	14.8 (3.3)
Follow-up time (y)	Mean (SD)	12.2 (4.6)	11.9 (5.1)
Cognitive status at baseline			
No cognitive impairment	*n*, %	95 (74.8)	47 (73.4)
Mild cognitive impairment	*n*, %	30 (23.6)	16 (25.0)
Possible/probable AD	*n*, %	2 (1.6)	1 (0.5)
Other dementia	*n*, %	0 (0.0)	0 (0.0)
Cognitive status at end of follow-up			
No cognitive impairment	*n*, %	40 (31.5)	19 (29.7)
Mild cognitive impairment	*n*, %	48 (37.8)	23 (35.9)
Possible/probable AD	*n*, %	35 (27.6)	22 (34.4)
Other dementia	*n*, %	4 (3.2)	0 (0.0)
Alzheimer’s disease	*n*, % who developed AD during follow-up	33 (26.0)	21 (32.8)
Mortality	*n*, % who died during follow-up (any cause)	38 (29.9)	18 (28.1)
	*n*, % who died during follow-up (with AD)	8 (6.3)	6 (9.4)
Time to development of AD (y)^a^	Mean (SD)	10.9 (6.1)	10.6 (5.6)
Age at death (y)^b^	Mean (SD)	84.9 (6.9)	85.1 (7.2)

^a^Among participants who developed AD during the study time frame (GM 6 − / − : *n* = 33, GM6 + / − or GM6 + / + : *n* = 21)

^b^Among participants who died during the study time frame (GM 6 − / − : *n* = 38, GM6 + / − or GM6 + / + : *n* = 18)

**Table 2:** Distribution of GM 3/17, GM 23, *HLA DRB1*, *FCGRIIB*, and *PILRA* genotypes in African American participants, stratified by GM 6 genotype

Variable	GM 6 − / − ( n = 127)	GM 6 + / − or GM 6 + / + ( n = 64)
GM 3/17 genotypes (%)		*
3/3	4.7	0.0
3/17	36.2	25.0
17/17	55.9	73.4
Untypable	3.2	1.6
GM 23 genotypes (%)		*
+ / +	66.9	85.9
+ / −	29.1	12.5
− / −	3.9	1.6
*HLA-DRB1* rs9271192 genotypes (%)		
AA	62.2	54.7
AC	31.5	40.6
CC	3.9	3.1
Untypable	2.4	1.6
*FCGRIIB* (%)		
CC	4.7	6.3
CT	40.9	35.9
TT	54.3	57.8
*PILRA* (%)		
AA	1.6	0.0
AG	27.6	29.7
GG	69.3	68.8
Untypable	1.6	1.6
*APOE* ε4 carrier positive (%)	33.1	40.6

*p < 0.05 when compared with GM 6 − / − by Fisher’s exact test

**Fig. 1:**
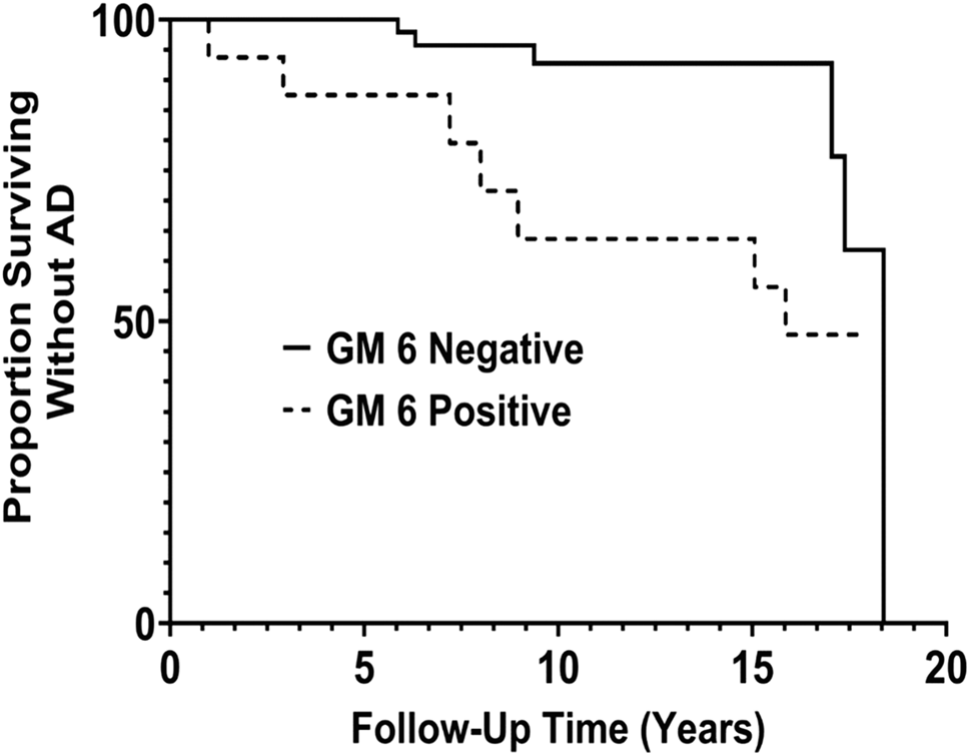
Proportion of African American participants without Alzheimer’s disease over time among participants with GM 3/17 or GM 3/3 (non-GM 17/GM 17), stratified by GM 6 status (HR of 4.63 in the results Table 3)

Table 3 presents the results of Cox proportional hazards modeling of time to development of AD as a function of GM 6 and other genes of interest. GM 6 by itself was not significantly associated with the development of AD (HR = 1.48; 95%CI 0.84, 2.61; *p* = 0.18). However, there was evidence of epistatic interaction: The risk of developing AD associated with GM 6 positivity was significantly different (*p* = 0.0098) in non-GM 17/GM 17 (i.e., GM 3/3 and GM 3/17) participants compared with GM 17/GM 17 participants. Specifically, in non-GM 17/GM 17 participants, the risk of AD was over fourfold higher in GM 6-positive participants compared with GM 6-negative participants (HR = 4.63; 95%CI 1.52, 14.1; *p* = 0.007), a finding that was not observed in GM17/GM17 participants (HR = 0.83, 95%CI 0.42, 1.66; *p* = 0.61). These results, depicted in Fig. 1, should be considered preliminary (i.e., hypothesis-generating), as the interaction *p*-value was not quite statistically significant after the FDR adjustment for multiple comparisons (adjusted *p*-value = 0.059).

**Table 3:** Association between GM 6 and time to development of AD, including interactions with other genes of interest

Proportional hazards model description*	Number of AD subjects/total number of subjects^†^	Hazard ratio for GM 6 positive vs. GM 6 negative**	95% confidence interval	*p*-value for main effect of GM 6	*p*-value for main effect of comparator gene	*p*-value for interaction term involving GM 6
GM 6	54/191	1.48	0.84–2.61	0.18	-	-
GM 6, GM 3/17, GM 6 × GM 3/17				0.61	0.0084	0.0098
GM 17/17	37/118	0.83	0.42–1.66			
GM 3/17 or GM 3.3	13/68	4.63	1.52–14.1			
GM 6, GM 23, GM 6 × GM 23				0.53	0.10	0.49
GM 23 − / −	43/140	1.22	0.65–2.30			
GM 23 + / − or GM 23 + / +	11/51	2.06	0.53–7.97			
GM 6, *HLA-DRB1*, GM 6 × *HLA-DRB1*				0.91	0.72	0.24
* HLA-DRB1* A	31/114	1.11	0.50–2.48			
* HLA-DRB1* A/C or HLA-C	22/73	1.75	0.75–4.09			
GM 6, *FCGRIIB*, GM 6 × FCGRIIB				0.69	0.96	0.07
* FCGRIIB* TT	27/106	0.83	0.35–2.01			
* FCGRIIB* CT or FCGRIIB CC	27/85	2.44	1.13–5.30			
GM 6, *PILRA*, GM 6 × *PILRA*				0.29	0.85	0.91
* PILRA* GG	35/132	1.45	0.73–2.89			
* PILRA* AG or *PILRA* AA	18/56	1.35	0.47–3.90			

*Models were Cox proportional hazards models, modeling the time to development of AD as a function of the genes of interest, each of which was coded as dichotomous variables, using a recessive modeling approach. All models controlled for the following covariates: baseline age, sex, education, APOE ε4

^†^A few of the subjects had specific genotypes that were untypable (see Table 2), which explains why the number of AD cases included in these models does not always total *n* = 54, and why the total number of subjects does not always total *n* = 191

**GM 6-positive includes GM 6 + / − and GM 6 + / + , while GM 6 negative is GM 6 − / −

**Fig. 2:**
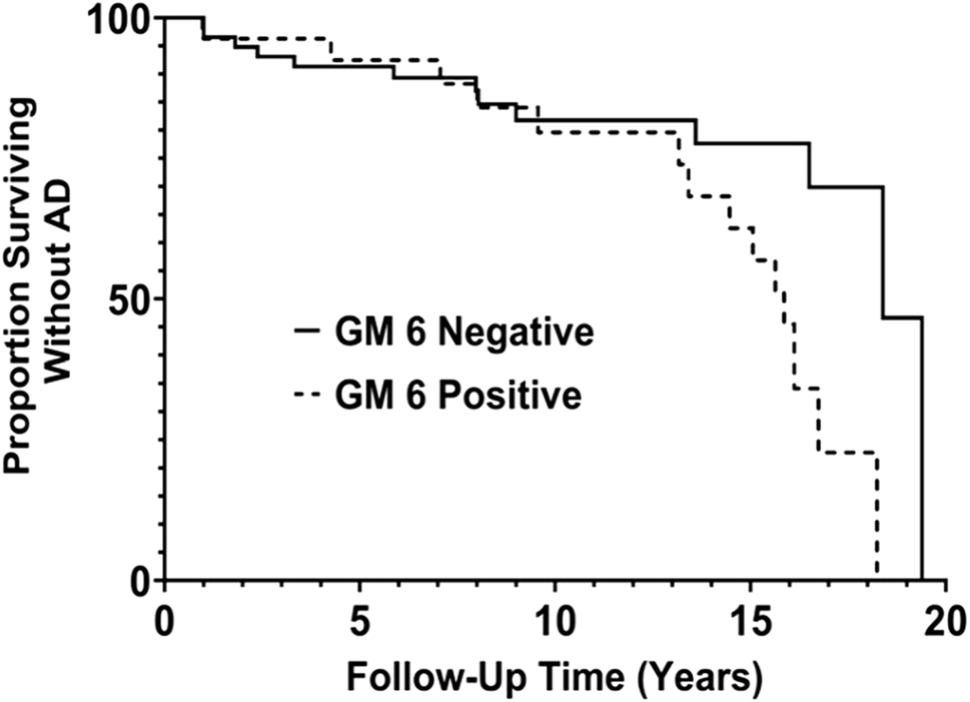
Proportion of African American participants without Alzheimer’s disease over time among participants with *FCGRIIB* CT or *FCGRIIB* CC (non-*FCGRIIB* TT), stratified by GM 6 status (HR of 2.44 in the results Table 3)

Similarly, risk of developing AD associated with GM 6 positivity was marginally but not significantly different (*p* = 0.07) in non-*FCGRIIB* TT participants compared with FCGRIIB TT participants. In non-*FCGRIIB* TT participants, the risk of developing AD was over twofold higher in GM 6-positive participants compared with GM 6-negative participants (HR = 2.44; 95%CI 1.13, 5.30; *p* = 0.02), a finding that was not observed in *FCGRIIB* TT participants (HR = 0.83; 95%CI 0.35, 2.01; *p* = 0.69). These results are depicted in Fig. 2.

## Discussion

The results presented here show that GM 6 may be a risk factor for the development of AD in an AA population, but only in the presence of certain other genes of the immune system. Participants who were positive for GM 6 (expressed on IgG3) and also expressed the GM 3 allele on IgG1 were at fourfold higher risk of developing AD. This association was independent of *APOE* ε4 genotype, the major risk factor for AD development. The exact mechanisms underlying this non-allelic/epistatic interaction are not clear, but one can speculate based on the known functional properties of GM allotypes. Since GM genes are associated with humoral immunity to a variety of self and nonself antigens (Oxelius and Pandey 2013; Warrender and Kelton 2020; Pandey and Li 2013), the mechanisms underlying their putative involvement in AD pathogenesis are most likely to include their role in antibody responsiveness to factors implicated in AD pathology. Several recent studies have provided strong evidence supporting a role for HSV1 in the etiopathogenesis of AD (Itzhaki 2018). HSV1 encodes a decoy Fcγ receptor (FcγR) which it uses to thwart the Fc-mediated effector functions, such as ADCC (Dubin et al. 1991). GM allotypes modulate this HSV1 immunoevasion strategy: IgG antibodies expressing different GM allotypes bind differentially to the decoy viral FcγR (Atherton et al. 2000). This provides a putative indirect mechanism for the GM gene involvement in the immunobiology of AD. GM allotypes are also associated with humoral immunity to several viruses, including herpesviruses (Biggar et al. 1984; Pandey et al. 2014b). GM 3 has been shown to be associated with higher anti-HSV1 antibody levels in a European AD cohort (Pandey et al. 2020). The influence of GM 6 on anti-HSV1 antibody responsiveness has not yet been investigated. It is relevant to note that GM 6 is extremely rare or absent in populations other than Africans, and, of the markers commonly present in this group, it has the most variable frequency, possibly a result of differential selection due to infectious pathogens (Steinberg and Cook 1981).

GM 6 also interacted with *FCGRIIB* alleles: In participants lacking the T allele of this gene, the risk of developing AD was over twofold higher in GM 6-positive participants compared with GM 6-negative participants. Homozygosity for the C allele of *FCGRIIB* has been shown to be associated with AD (Pandey et al. 2021). Thus, this locus has both independent and epistatic (with GM 6) effect on the development of AD. In animal models of AD, *FCGRIIB* is associated with neuronal uptake and inter-neuronal accumulation of Aβ (Gwon et al. 2018). It is possible that there is a greater accumulation of Aβ in people carrying the C allele of *FCGRIIB* and anti-Aβ IgG3 autoantibodies expressing the GM 6 allotype in the Fc region of these antibodies have a low affinity for the activating receptor FcγRIIa expressed on microglia. This would reduce the level of antibody-dependent microglia-induced phagocytosis of Aβ, and thus contribute to AD development. Studies to determine the possible contribution of GM 6 to antibody-dependent phagocytosis of Aβ are in progress.

## Conclusions

In conclusion, IgG3 allotype GM 6, which is present almost exclusively in people of African descent, may be a risk factor for AD development when accompanied by particular *IGHG* and *FCGRIIB* alleles. This study underscores the importance of epistasis, which is usually not measured in GWAS and other genomic studies. Such gene–gene interaction studies could shed light on the “missing” heritability in AD and other polygenic multifactorial diseases. The results presented here need to be replicated in independent and larger studies.

## Data Availability

Data generated in this study will be available from the corresponding author upon reasonable request.
